# Adaptive functioning in children and young adults with monogenic neurodevelopmental disorders

**DOI:** 10.1111/dmcn.16227

**Published:** 2025-01-23

**Authors:** Emma K. Baker, Miya St John, Ruth Braden, Lottie D. Morison, Elana J. Forbes, Fatma Lelik, Stephen J. C. Hearps, David J. Amor, Angela T. Morgan

**Affiliations:** ^1^ Speech and Language Murdoch Children's Research Institute Parkville Victoria Australia; ^2^ Department of Paediatrics University of Melbourne Melbourne Victoria Australia; ^3^ School of Psychology and Public Health La Trobe University Melbourne Victoria Australia; ^4^ Department of Audiology and Speech Pathology University of Melbourne Melbourne Victoria Australia; ^5^ Brain and Mind Murdoch Children's Research Institute Parkville Victoria Australia; ^6^ Department of Critical Care University of Melbourne Melbourne Victoria Australia; ^7^ Neurodisability and Rehabilitation Murdoch Children's Research Institute Parkville Victoria Australia; ^8^ Speech Pathology Department Royal Children's Hospital Parkville Victoria Australia

## Abstract

**Aim:**

To examine the adaptive behaviour profiles of children with monogenic neurodevelopmental disorders (NDDs) to determine whether syndrome‐specific or transdiagnostic approaches provide a better understanding of the adaptive behavioural phenotypes of these NDDs.

**Method:**

This cross‐sectional study included parents and caregivers of 243 (48% female) individuals (age range = 1–25 years; mean = 8 years 10 months, SD = 5 years 8 months) with genetically confirmed monogenic NDDs (*CDK13*, *DYRK1A*, *FOXP2*, *KAT6A*, *KANSL1*, *SETBP1*, *BRPF1*, and *DDX3X*). Parents and caregivers completed the Vineland Adaptive Behavior Scales, Third Edition to assess communication, daily living, socialization, and motor skills.

**Results:**

Linear regression models comparing mean adaptive behaviours between monogenic NDDs, adjusting for the presence of intellectual disability, revealed few group differences. Children with variants in *BRPF1* or *KANSL1* had better adaptive behaviour skills compared to children with variants in *CDK13*, *DDX3X*, *DYRK1A,* and *KAT6A*, although group differences varied across domains. A latent profile analysis showed compelling evidence for a five‐profile model. These profiles were homogeneous, with similar delays across the subdomain scores in each profile. Additionally, each monogenic NDD was represented in each profile, with a few exceptions.

**Interpretation:**

Transdiagnostic approaches to understand adaptive behaviour in monogenic NDDs provide a better understanding of individual strengths and challenges, enabling more targeted support.

## Graphical abstract

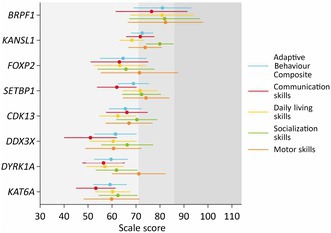

Plain language summary: https://onlinelibrary.wiley.com/doi/10.1111/dmcn.16264

**Abbreviations**
ABC: Adaptive Behaviour Composite
LPA: latent profile analysis
NDD: neurodevelopmental disorder
Vineland‐3: Vineland Adaptive Behavior Scales, Third Edition

**What this paper adds**

- There was no pattern of adaptive strengths and challenges in each syndrome.
- Syndrome‐specific profiles were absent, regardless of intellectual abilities.
- Profile analysis emphasized the diversity in adaptive behaviours in each monogenic condition.
- Transdiagnostic approaches emphasize individual strengths and challenges, regardless of the monogenic condition.

Neurodevelopmental disorders (NDDs) alter neurological development and influence how the brain develops, causing difficulties in cognitive, social, and emotional functioning.^1^ Children with NDDs present with varying degrees of intellectual disability, speech and language disorders, motor deficits, autistic traits, attention difficulties, and adaptive behaviour challenges.^2^ Adaptive behaviours are skills that are required for independent living and include communication, daily living, and social skills. For individuals with NDDs, understanding these skills can be important for developing targeted interventions to increase the likelihood of independent living as adults.

Deep neurobehavioural phenotyping is currently lacking for most monogenic NDDs, with specific adaptive profiles often overshadowed by intellectual disability. While some argue for the presence of syndrome‐specific adaptive behaviour profiles to enable targeted interventions,^3^ others suggest that such categorical approaches overlook the clinical heterogeneity in and across diagnostic categories and impede the ability to identify individual strengths and risk factors, understand the underlying mechanisms, and find appropriate support systems.^4^ Furthermore, the specific co‐occurring conditions experienced by each individual likely affect adaptive behaviour profiles. For example, co‐occurring autism is associated with lower socialization skills relative to other adaptive domains in both polygenic and monogenic conditions with and without intellectual disability,^5^, ^6^, ^7^ while speech and language disorders are commonly associated with lower communication skills.^8^, ^9^ Nonetheless, research including individuals with several genetic syndromes has demonstrated the unique impact of speech and language challenges on broader adaptive behaviour domains. A recent study^10^ in individuals with Down syndrome showed scores on the Speech Scale of the Communication Checklist, Second Edition to be the only significant predictor of global, conceptual, and practical adaptive behaviours, as assessed by the Adaptive Behavior Assessment System, Second Edition.

Research that compares adaptive behaviour profiles across different monogenic NDDs is lacking. While categorical approaches aim to include homogeneous samples of individuals, it is widely accepted that in each NDD a large amount of phenotypic variability exists. In a recent study, Kreemers et al.^4^ examined adaptive behaviour profiles using the Adaptive Behavior Assessment System, Third Edition in a large sample (*n* = 222) of children with autism or intellectual disability from both categorical and transdiagnostic approaches. Children with only autism showed weaker social skills, relative to other domains, while those in the intellectual disability only and autism plus intellectual disability groups showed weaker conceptual skills compared to social and practical skills. Nonetheless, a profile analysis revealed three profiles characterized by: (1) an absence of strengths and weaknesses (homogeneous profile); (2) relatively strong social and leisure skills with weaker self‐care skills (social profile); and (3) relatively strong academic and community skills with relatively weak communication, social, and self‐care skills (academic profile). Within these profiles, the authors noted two key findings: most participants (*n* = 139; 63%) fell in the homogeneous profile; and there were no significant differences between different diagnostic classifications, suggesting that an individualized approach, regardless of the diagnostic category, may be more beneficial. Moreover, understanding the patterns of strengths and weaknesses within and across conditions is of value for targeted clinical management.

Individuals included in this study were those with a pathogenic variant in *CDK13*, *DYRK1A*, *FOXP2*, *KAT6A*, *KANSL1*, *SETBP1*, *BRPF1*, or *DDX3X*. These conditions vary in the degree of intellectual abilities, behavioural challenges, and presence of co‐occurring conditions. Notably, the conditions included in this study all have prominent speech and language profiles. Of note, most individuals with variants in *DDX3X*, *KAT6A*, and *DYRK1A* have moderate‐to‐severe intellectual disability and do not typically use verbal speech to communicate. In addition, individuals with variants in *DYRK1A* are more vulnerable to the development of epilepsy and seizures and are often reported to have co‐occurring autism.^11^ Individuals with variants in *BRPF1* and *FOXP2* typically have milder intellectual disability and usually use speech to communicate, although phonological delay is common in individuals with *BRPF1* variants^12^ and childhood apraxia of speech is a key characteristic of children with *FOXP2* variants.^13^ Individuals with variants in *KANSL1*, *SETBP1*, and *CDK13* have intellectual disability that falls within the mild‐to‐moderate range. Epilepsy is also common in those with variants in *KANSL1*.^14^ Nonetheless, across all of these syndromes, there is variability in clinical phenotype, with some individuals not sharing all characteristics (including the level of intellectual disability) of the particular syndrome.

This large cohort of children and young adults with monogenic NDDs, ascertained based on speech and language challenges, was used to delineate the presence (or absence) of syndrome‐specific profiles and to determine whether transdiagnostic approaches to understand behavioural phenotypes in monogenic conditions may provide a more nuanced understanding of strengths and challenges.

## METHOD

### Participants

This cross‐sectional study included a convenience sample of 243 (48% female) children and young people with 1 of 8 genetically confirmed monogenic NDDs (pertaining to variants in *CDK13*, *DYRK1A*, *FOXP2*, *KAT6A*, *KANSL1*, *SETBP1*, *BRPF1*, and *DDX3X*) aged 1 to 25 years (mean = 8 years 10 months, SD = 5 years 8 months). Families were recruited nationally and internationally through the National Health and Medical Research Council (Australia) Centre of Research Excellence in Speech and Language, as part of broader cohort studies of these conditions from January 2019 to December 2023. These broader ‘reverse phenotyping’ cohort studies aimed to provide comprehensive phenotyping of the speech and language profiles of several genetic conditions.^12^, ^15^, ^16^, ^17^, ^18^, ^19^, ^20^, ^21^ Individuals were included in these previous studies if they had a confirmed molecular diagnosis and excluded if they had any other confirmed genetic variant or syndrome that was likely to affect the clinical phenotype.

Participants were included in the current study based on having a completed Vineland Adaptive Behavior Scales, Third Edition (Vineland‐3)^22^ and aged between 1 year and 25 years. Ethics approval was obtained from the Royal Children's Hospital Human Research Ethics Committee (no. HREC37353). Informed written consent was provided by caregivers and legal guardians.

### Data collection

Parents completed an online health and medical questionnaire to collect demographic information and determine the presence of co‐occurring conditions, such as intellectual disability, autism spectrum disorder (hereafter ‘autism’), and attention‐deficit/hyperactivity disorder (ADHD). Response options for autism and ADHD were ‘yes’ or ‘no’, while for intellectual disability the response options were, ‘yes’, ‘no’, or ‘not applicable’ (too young or not tested). When indicated to be present, parents and caregivers uploaded copies of original clinical assessment reports, if applicable, to confirm the presence or absence of the condition (intellectual disability, autism, and ADHD). In some instances, no formal assessment was completed but parents indicated the presence or absence of the condition. For other individuals, no formal assessment or parent report of a diagnosis was provided. For these individuals, it was assumed that there was no indication or parental concern of autism or ADHD that warranted assessment; thus, the condition was classified as ‘absent’. To enable classification of intellectual disability in these individuals, review of the health and medical history form, including age at attaining developmental milestones, presence or absence of developmental delay, and letters from medical or allied health professionals, was undertaken by the first author (EKB) to provide a ‘clinician‐inferred’ presence or absence of intellectual disability.

Parents completed the Vineland‐3 comprehensive parent form^22^ to assess communication, daily living, socialization, and motor skills, and their respective subdomains. The Vineland‐3 was completed in the participants' preferred language (English, Spanish, Dutch, or German) through an online research data collection platform (REDCap, Vanderbilt University, Nashville, TN, USA)^23^, ^24^ hosted at the Murdoch Children's Research Institute, with permission to reproduce the Vineland‐3 on REDCap from Pearson Clinical Assessments Australia. Scaled scores for the Adaptive Behaviour Composite (ABC) and the Vineland‐3 domains were calculated, as were the v‐scaled scores for the subdomains. Scaled scores ranged from 20 to 140, with a mean of 100 and SD of 15, while v‐scaled scores ranged from 1 to 24, with a mean of 15 and SD of 3. Qualitative descriptors are also provided for ranges of scaled scores (20–70 = low; 71–85 = moderately low; 86–114 = adequate; 115–129 = moderately high; 130–140 = high) and v‐scaled scores (1–9 = low; 10–12 = moderately low; 13–17 = adequate; 18–20 = moderately high; 21–24 = high).

### Statistical analysis

Demographic (age and sex) and clinical characteristics (intellectual disability, autism, and ADHD) were summarized between diagnostic groups. Frequencies and percentages were used for categorical variables and means and SDs for child age. Linear regression models compared mean adaptive behaviour skills between diagnostic groups, adjusting for the presence of intellectual disability. Model‐adjusted means and 95% confidence intervals (CI) were plotted; post hoc Bonferroni‐corrected comparisons were made between each group. Overall, data missingness was low for adaptive behaviour skills outcomes (4.9%)^25^ and results presented using all available data.

To examine potential underlying adaptive behaviour skills profiles in the group, latent profile analysis (LPA) was conducted on the total sample. LPA is a mixture model used to determine latent groups of a sample from observed variable patterns.^26^ Observed v‐scaled scores for the subdomains of communication, daily living, and social skills were standardized, and the LPA of these with two to seven profiles were compared for optimal fit. Model fit was assessed using the Akaike and the Bayesian information criteria, then the Vuong–Lo–Mendell–Rubin adjusted likelihood ratio test and entropy. A better model fit was indicated by lower Akaike and Bayesian information criteria values, with emphasis placed on a change (Δ) in Bayesian information criteria greater than 10, that is, ‘strong evidence’ for improvement,^27^ a significant Vuong–Lo–Mendell–Rubin *p*‐value, and entropy greater than 0.8 (with values closer to 1 being optimal).^26^ Demographic and clinical characteristics, including autism, ADHD, and intellectual disability were again compared between the derived profiles. Statistical significance was set at *p* < 0.05 for all analyses; all analyses were carried out using Stata v18.0 (StatCorp, College Station, TX, USA).^28^

## RESULTS

### Co‐occurring conditions

Of the 243 participants, 73 (30%) had a confirmed intellectual disability based on documented clinical assessment. A further 61 (25%) individuals were reported by their parent or caregiver to have an intellectual disability, and 48 (20%) had a clinician‐inferred intellectual disability. Only 21 (9%) participants were confirmed to have no intellectual disability based on documented clinical assessment, while a further 21 (9%) were reported by their parents to have no intellectual disability, and 8% were clinician‐inferred as not having an intellectual disability. Overall, 182 (75%) of the children and young adults in this study were deemed to have an intellectual disability.

Regarding the presence of other co‐occurring NDDs, 32 (13%) participants had a confirmed diagnosis of autism, with a further 17 (7%) reported to have a diagnosis of autism by their parent or caregiver. Similar rates of ADHD were endorsed, with 24 (10%) participants confirmed to have a diagnosis of ADHD and a further 14 (6%) with a diagnosis of ADHD according to parent report.

For most of the genetic conditions, the presence of intellectual disability was confirmed or parent‐reported, except for individuals with the *BRPF1* or *FOXP2* variants (Table 1). Most children were not indicated as having autism or ADHD, except for those with a *DYRK1A* variant, where almost 50% of children either had a confirmed diagnosis or parent report of autism. Information relating to the age, biological sex, intellectual ability, and presence of other co‐occurring conditions (autism and ADHD) are provided in Table 1.

**TABLE 1:** Demographic information and co‐occurring conditions for each monogenic condition.

Demographic characteristic/co‐occurring condition	Gene							
	*BRPF1*	*KANSL1*	*FOXP2*	*SETBP1*	*CDK13*	*DDX3X*	*DYRK1A*	*KAT6A*
*n*	11	66	17	33	32	19	31	34
Age, mean (SD), years:months	6:7 (3:10)	9:6 (6:0)	10:2 (6:11)	8:10 (4:11)	8:6 (5:4)	7:1 (4:0)	10:4 (6:11)	7:6 (4:7)
Sex, % female	18	41	29	45	59	100	39	50
Intellectual disability, %								
Intellectual disability confirmed	9	35	18	55	22	16	23	32
Intellectual disability parent‐reported	0	24	12	15	6	58	55	24
Intellectual disability clinician‐inferred	9	24	0	6	19	26	19	35
No intellectual disability confirmed	27	0	41	9	19	0	3	3
No intellectual disability parent‐reported	18	8	24	9	22	0	0	0
No intellectual disability clinician‐inferred	36	9	6	6	13	0	0	6
Autism, %								
Present (assessed)	9	5	29	15	9	0	22	24
Present (parent‐reported)	0	1	6	0	9	21	26	0
Not present	91	94	65	85	82	79	52	76
ADHD, %								
Present (assessed)	0	9	0	33	3	0	6	12
Present (parent‐reported)	18	2	6	0	9	0	23	0
Not present	82	89	94	67	88	100	71	88

Abbreviation: ADHD, attention‐deficit/hyperactivity disorder.

### Syndrome‐specific profiles and group comparisons of adaptive behaviour skills

**FIGURE 1:**
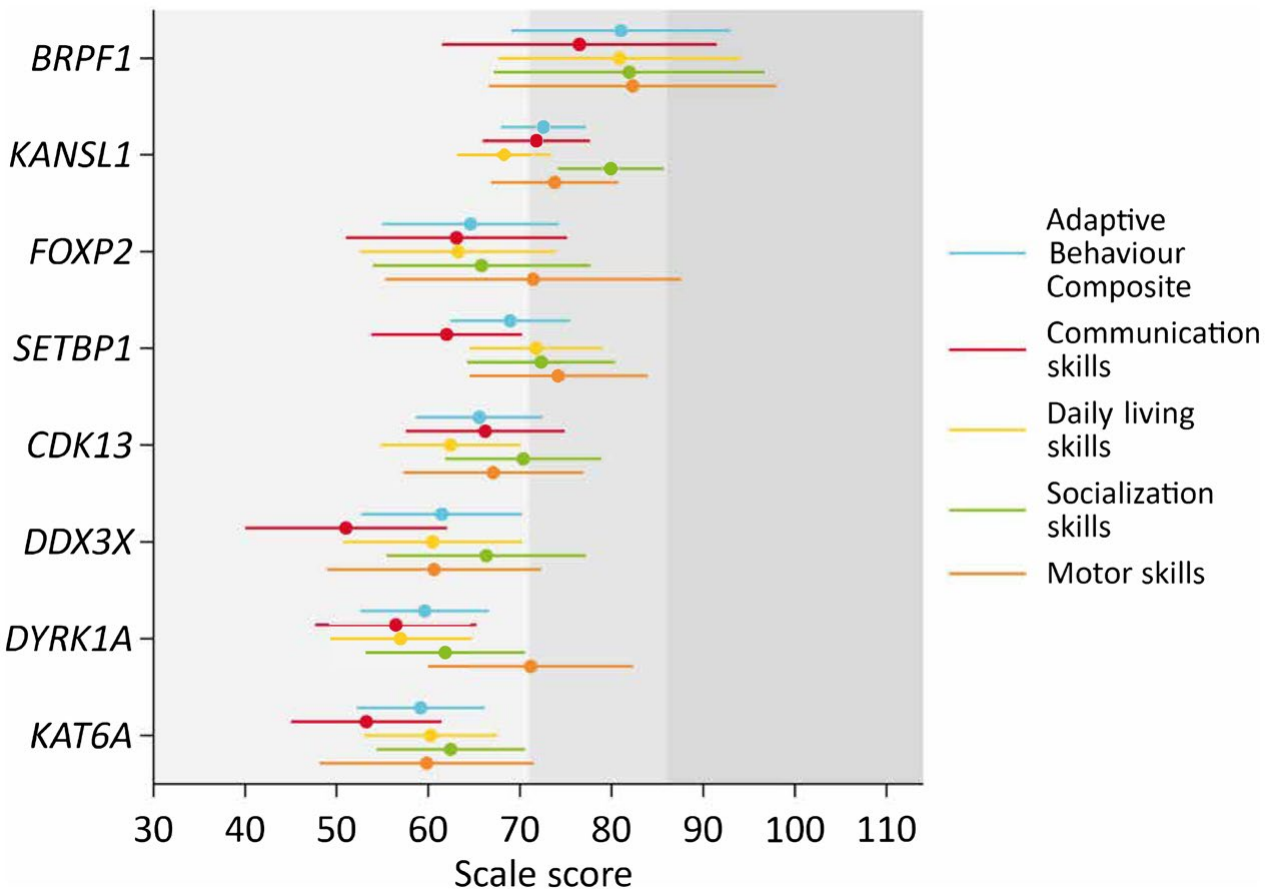
Model‐estimated (intellectual disability‐adjusted) adaptive behaviour domain scaled score means and 95% confidence intervals. The dark grey shading indicates the adequate range; the light grey shading indicates the moderately low range; the lightest grey shading indicates the low range.

Examining the adaptive behaviour profiles of each individual syndrome, skills were homogeneous across the domains assessed (Figure 1). For most syndromes, socialization (*KANSL1*, *CDK13*, *DDX3X*, *KAT6A*) or motor skills (*FOXP2*, *SETBP1*, *DYRK1A*) were the strongest skillset in the profile, while communication skills were the weakest, except for the *KANSL1* and *CDK13* groups where daily living skills were the weakest. Means and SDs for the ABC, domain scores, and subdomain scores for each genetic syndrome are provided in Table S1.

Few group differences existed when comparing the domain scores of the Vineland‐3, adjusting for intellectual disability. The *BRPF1* group had significantly (post hoc pairwise Bonferroni comparisons) higher ABC and daily living scores compared to the *CDK13* (ABC: mean difference [M_diff_] = 15.5, 95% CI = 0.3–30.6; daily living: M_diff_ = 18.4, 95% CI = 1.5–35.3), *DDX3X* (ABC: M_diff_ = 19.5, 95% CI = 2.1–37.0; daily living: M_diff_ = 20.4, 95% CI = 1.0–39.8), *DYRK1A* (ABC: M_diff_ = 21.4, 95% CI = 5.1–37.7; daily living: M_diff_ = 23.9, 95% CI = 5.8–42.0), and *KAT6A* (ABC: M_diff_ = 21.8, 95% CI = 5.5–38.2; daily living: M_diff_ = 20.6, 95% CI = 2.9–38.3) groups. The *BRPF1* group also had significantly higher communication scores compared to the *DDX3X* (M_diff_ = 25.5, 95% CI = 3.6–47.4) and *KAT6A* (M_diff_ = 23.3, 95% CI = 3.2–43.3) groups (Figure 1).

The *KANSL1* group had higher ABC, communication, and socialization scores than the *DYRK1A* (ABC: M_diff_ = 13.0, 95% CI = 3.5–22.5; communication: M_diff_ = 15.3, 95% CI = 3.4–27.3; socialization: M_diff_ = 18.1, 95% CI = 6.3–29.9) and *KAT6A* (ABC: M_diff_ = 13.4, 95% CI = 3.9–22.9; communication: M_diff_ = 18.5, 95% CI = 7.1–30.0; socialization: M_diff_ = 17.5, 95% CI = 6.2–28.7) groups. Additionally, the *KANSL1* group had higher communication scores compared to the *DDX3X* (M_diff_ = 20.8, 95% CI = 6.6–34.9) group, and higher daily living scores compared to the *DYRK1A* (M_diff_ = 11.3, 95% CI = 0.7–21.9) group. The *SETBP1* group had higher daily living skills compared to the *DYRK1A* (M_diff_ = 14.8, 95% CI = 2.6–27.0) group. No other statistically significant between‐group differences were detected. The pattern of group differences was similar for the subdomain scores (Figure S1).

### Transdiagnostic approach to adaptive behaviour profiles

**FIGURE 2:**
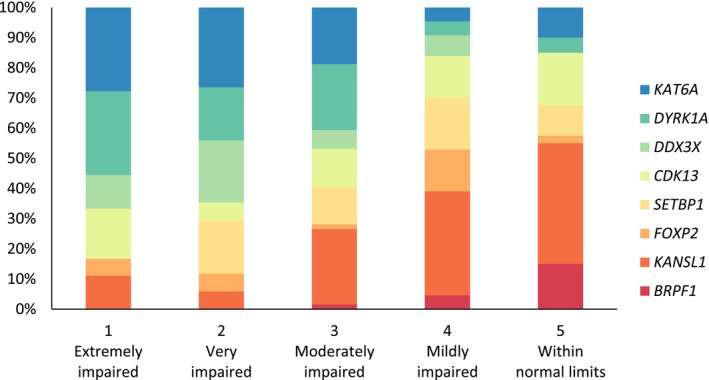
Distribution of monogenic neurodevelopmental disorders within latent profiles.

An LPA compared two to five latent profile models and showed robust evidence for a five‐profile model as the best model fit (Table S2). The profiles were homogeneous, with similar delays across the subdomain scores (Table 2). Consequently, profiles were labelled ‘extremely impaired’ (profile 1), ‘very impaired’ (profile 2), ‘moderately impaired’ (profile 3), ‘mildly impaired’ (profile 4), and ‘within normal limits’ (profile 5). Examining the genetic conditions that fell within each profile revealed within‐syndrome heterogeneity, with most conditions having individuals falling within each profile (Figure 2), the exceptions being *BRPF1* (no individuals in profiles 1 and 2), *SETBP1* (no individuals in profile 1), and *DDX3X* (no individuals in profile 5).

**TABLE 2:** Comparison of latent profiles.

	Profile 1			Profile 2			Profile 3			Profile 4			Profile 5		
	*n* ^a^	*n* ^b^	%	*n* ^a^	*n* ^b^	%	*n* ^a^	*n* ^b^	%	*n* ^a^	*n* ^b^	%	*n* ^a^	*n* ^b^	%
Sex															
Female	18	8	44.4	34	18	52.9	64	33	51.6	87	39	44.8	40	18	45.0
Male	18	10	55.6	34	16	47.1	64	31	48.4	87	48	55.2	40	22	55.0
Autism															
Present (assessed)	18	3	16.7	34	9	26.5	64	10	15.6	87	8	9.2	40	2	5.0
Present (parent‐reported)	18	3	16.7	34	7	20.6	64	5	7.8	87	2	2.3	40	0	0
Not present	18	12	66.7	34	18	52.9	64	49	76.6	87	77	88.5	40	38	95.0
ADHD															
Present (assessed)	18	0	0	34	2	5.9	64	7	10.9	87	11	12.6	40	4	10.0
Present (parent‐reported)	18	2	11.1	34	3	8.8	64	4	6.3	87	4	4.6	40	1	2.5
Not present	18	16	88.9	34	29	85.3	64	53	82.8	87	72	82.8	40	35	87.5
Intellectual abilities															
High average	16	0	0	22	1	4.5	43	0	0	51	0	0	20	0	0
Average	16	0	0	22	0	0	43	0	0	51	1	2.00	20	2	10.0
Low average	16	0	0	22	0	0	43	0	0	51	3	5.9	20	3	15.0
Borderline	16	0	0	22	1	4.5	43	1	2.3	51	4	7.8	20	0	0
Mild	16	4	25.0	22	0	0	43	10	23.3	51	17	33.3	20	5	25.0
Moderate	16	5	31.2	22	12	54.6	43	23	53.5	51	22	43.1	20	8	40.0
Severe	16	7	43.8	22	8	36.4	43	9	20.9	51	4	7.8	20	2	10.0

	*n*	Mean	SD	*n*	Mean	SD	*n*	Mean	SD	*n*	Mean	SD	*n*	Mean	SD
Age, years:months	18	11:4	4:10	34	8:4	5:2	64	8:2	5:5	87	9:0	6:1	40	8:11	5:7
Vineland‐3 raw v‐scores															
Receptive communication	18	2.3	2.6	34	3.4	3.1	64	8.1	2.2	87	11.0	2.0	40	14.0	1.8
Expressive communication	18	1.8	2.5	34	2.7	2.9	64	7.1	3.3	87	10.2	2.9	40	13.6	2.8
Written communication	18	2.0	1.4	33	5.9	3.3	55	6.6	2.8	73	9.0	3.6	37	11.5	3.4
Personal daily living	17	1.9	1.6	33	3.8	2.6	63	6.7	2.1	85	10.3	2.4	40	12.3	2.6
Domestic daily living	17	4.6	1.8	32	7.0	2.2	53	8.4	2.1	72	10.8	2.1	37	12.8	2.2
Community daily living	17	2.1	1.0	32	5.1	1.9	53	7.2	1.8	72	9.5	1.6	37	12.2	2.6
Interpersonal relationship	17	2.5	1.5	33	5.6	1.5	63	8.4	1.9	85	11.3	1.5	40	14.5	1.8
Play and leisure	17	1.7	1.4	33	5.0	2.4	63	9.1	1.8	85	11.5	1.6	40	14.2	1.8
Coping and adapting	17	4.0	1.6	33	7.0	1.7	58	9.0	1.7	80	11.2	1.8	38	14.1	1.4

^a^ Profile frequency.

^b^ Within‐profile positive frequency.

Abbreviations: ADHD, attention‐deficit/hyperactivity disorder; Vineland‐3, Vineland Adaptive Behavior Scales, Third Edition.

The contribution of autism, ADHD, and intellectual disability to the specific LPA profiles was examined. A higher proportion of individuals with co‐occurring autism were in the extremely and moderately impaired profiles compared to the mildly delayed and within normal limits profiles. No specific pattern for ADHD was observed across the profiles. Regarding intellectual disability, a higher proportion of individuals with intellectual disability in the low average and average IQ ranges were more likely to be in the mildly impaired and within normal limits profiles, although exceptions were present. Two individuals with severe intellectual disability were reported to be in the within normal limits profile, while an individual with high average IQ was in the moderately impaired profile. This individual also had a confirmed diagnosis of autism and their parent reported that they required a significant amount of prompting to perform adaptive behaviour tasks.

## DISCUSSION

This study aimed to delineate whether specific adaptive behaviour profiles were evident in eight different monogenic NDDs ascertained based on speech and language challenges. Although some nuanced patterns of strengths and weaknesses were observed in some of the conditions, mostly relating to communication (stronger receptive communication skills in *SETBP1* and *FOXP2* relative to expressive and written communication skills; weaker overall communication skills in *SETBP1* and *DDX3X* relative to other domains; and weaker written communication skills in *KANSL1* and *CDK13*), no obvious specific profiles were shown. When comparing scores between groups adjusting for the presence of intellectual disability, only *BRPF1* and *KANSL1* significantly differed from the *DYRK1A*, *KAT6A*, and *DDX3X* groups on the Vineland‐3 domains; other statistically significant group differences were not detected between the groups, further suggesting a lack of distinct syndrome‐specific profiles beyond the level of intellectual disability.

A recent review by Astle et al.^29^ further emphasized that transdiagnostic approaches may also help to identify the barriers that children with NDDs encounter, aid understanding of the underlying mechanisms, and find the most appropriate and effective pathways to intervention. Using clustering approaches, Kushki et al.^30^ demonstrated distinct clusters of children with varying polygenic NDDs (autism, ADHD, and obsessive‐compulsive disorder) based on both questionnaire and neural (cortical thickness) data. Each diagnostic group was represented across each cluster with no significant alignment of diagnostic classification using the data‐driven grouping approach. Kushki et al.^30^ concluded that existing diagnostic labels may not capture aetiologically, biologically, and phenomenologically homogeneous groups. In a recent study, Brkić et al.^31^ contrasted NDDs with a known genetic cause, based on the functional network membership of the genetic variant on several autistic traits. Individuals with genetic variants involved in synaptic transmission, synapse‐associated cytoskeleton, or postsynaptic intracellular signalling were allocated to the ‘synaptic’ group, while individuals with variants in genes associated with chromatin structural regulation were allocated to the ‘chromatin’ group. The results demonstrated that those in the chromatin group had higher inflexibility levels compared to those in the synaptic physiology group, although no other traits of autism were predicted by gene function.

In those NDDs where there is a known genetic cause and the causative gene has a known biological or cellular function, these NDDs may be grouped functionally.^29^ In the current study, three of the monogenic NDDs are caused by genes that are associated with chromatin regulation (*BRPF1*, *KANSL1*, and *KAT6A*), while the remaining conditions are associated with transcriptional processes. Interestingly, *BRPF1* and *KANSL1* were typically associated with stronger adaptive profiles compared to the other conditions. Yet, *KAT6A* had weaker adaptive abilities, particularly compared to *BRPF1* and *KANSL1*, suggesting that gene functions still may not accurately categorize the behavioural phenotypes of monogenic NDDs.

One study that compared the adaptive behaviours of several monogenic NDDs (Kleefstra syndrome, Koolen‐de Vries syndrome, and *GATAD2B*‐related syndrome, and a mixed group) advocated for syndrome‐specific adaptive and maladaptive functioning.^3^ Nonetheless, examination of the adaptive profiles revealed consistency with the findings of the current study. Individuals with Kleefstra syndrome and *GATAD2B*‐related syndrome, which are both typically associated with moderate‐to‐severe intellectual disability, had lower adaptive profiles compared to the Koolen‐de Vries syndrome group (typically mild intellectual disability). Differences were observed across maladaptive profiles with more co‐occurring mental health conditions in those with Kleefstra syndrome, with elevated rates of autism, current major depressive disorder, current obsessive‐compulsive disorder, and current and past psychosis, which may also be contributing to lower adaptive skills in this group. Nonetheless, co‐occurring conditions were also common across the other two groups: anxiety and ADHD in Koolen‐de Vries syndrome, and sleep problems, anxiety, and mood disorders in *GATAD2B*‐related syndrome. Our LPA findings also demonstrated the impact of co‐occurring autism, but not ADHD, on the specific adaptive behaviour profiles of individuals with genetic NDDs. Thus, consideration of co‐occurring conditions is critical to enable targeted treatments, which may also have positive impacts on adaptive behaviour profiles.

While this study has specifically focused on conditions identified based on their speech and language profiles, the work has implications across the spectrum of NDDs. While disorder‐specific assessments that capture nuances of each condition are frequently pursued, the findings presented in this study provide support for transdisciplinary assessments that are developed for a wide range of NDDs. Most assessment tools used in the NDD field were developed based on typical development or general clinical populations, and thus fail to capture the nuances of NDDs broadly. These tools also lack assessment of the modifications and accommodations that may be in place for each individual. For example, the use of augmentative and alternative communication (AAC) systems has led to improved adaptive behaviour skills, with AAC supporting communication and socialization skills, and decreasing frustration and maladaptive coping mechanisms;^32^, ^33^ yet, the most widely used tools in the NDD field do not capture the use of such systems. One tool that has aimed to capture communicative functioning is the Communication Function Classification System.^34^ The Communication Function Classification System was developed for use in cerebral palsy but it is now used across a wide range of conditions. Including features of the Communication Function Classification System in newly developed tools would be beneficial. New NDD‐specific tools should aim to capture both the strengths and challenges in each individual profile, and consider the presence of co‐occurring conditions and any accommodations that the individual may use to facilitate adaptive behaviour. Such tools would provide a better pathway towards effective treatments for each individual that optimize their functioning and participation.

### Strengths and limitations

While this is one of the largest studies to compare the adaptive behaviour profiles of several monogenic disorders, several limitations exist. One of the primary limitations is the biased ascertainment of the conditions included. All cohorts were ascertained for speech and language research. Consequently, individuals referred to the study may have been more likely to have speech and language profiles more severe than might be expected for the wider population of those with the specific syndrome and contributed to lower scores on the Vineland‐3, particularly on the communication domain. Having only one measure of behaviour also limited the ability to examine other specific profiles that may (or may not) have emerged in particular syndromes. In future research, inclusion of additional measures of behaviour, executive functioning, communication skills, and co‐occurring neurodevelopmental and mental health conditions would provide further insights into the common challenges experienced across NDDs, and whether profiles are like those presented in this study or change when accounting for these other factors.

The use of parent and caregiver reports, and clinician‐inferred diagnoses of intellectual disability, limited the ability to examine the effect of intellectual disability on adaptive behaviour profiles for the included conditions. Nonetheless, profiles were consistent with what is expected based on previous literature, with conditions associated with no or milder intellectual disability having stronger adaptive behaviour profiles than conditions associated with more severe intellectual disability. Similarly, diagnoses of autism and ADHD were confirmed through diagnostic reports for a small sample of children, with most children across cohorts not reported by their parents to have these co‐occurring conditions. Given that the primary research studies, from which the cohorts were drawn, aimed to characterize the speech and language phenotype, further assessment was not undertaken for those individuals who were not indicated by their parents to have co‐occurring conditions. Therefore, analyses relating to the impacts of co‐occurring autism and ADHD should be interpreted with caution. It is possible that some individuals who were not reported to have autism or ADHD by their caregiver may indeed meet the diagnostic criteria for these conditions. The complex nature of the syndromes included in the current study may also lead parents not to seek additional diagnoses because of diagnostic overshadowing, difficulty accessing diagnostic services, or there being minimal change in clinical care if their child were to receive a co‐occurring diagnosis. In contrast, parent‐reported diagnoses may represent traits of these conditions, but not meet the diagnostic criteria. Nonetheless, our findings are in line with previous research, demonstrating greater impacts on adaptive behaviour when a child has co‐occurring autism.^5^, ^6^

Additionally, smaller samples for some of the monogenic conditions (e.g. the *BRPF1*, *FOXP2*, and *DDX3X* variants) may not be entirely reflective of the population of individuals with these variants. Individuals included in this study may be biased, with parents of children with more complex needs expressing interest in research to better understand their child's strengths and challenges.

### Conclusions

Both polygenic and monogenic NDDs are heterogeneous in their clinical presentations. Current approaches to categorizing NDDs do not accurately capture this degree of variability and therefore the specific needs of the individual. Additionally, categorical approaches fail to consider the overlap in symptoms across the NDD spectrum. Transdiagnostic frameworks for identification and intervention across NDDs provide a child‐centred approach that can enable targeted support for the most impactful characteristics, rather than diagnostic‐driven characteristics.^4^ The present results provide further evidence to support such frameworks, due to the lack of syndrome‐specific adaptive profiles and representation of distinct syndrome groups across putative latent groupings. Movement towards needs‐based assessments can identify areas for targeted support, building from areas of relative strength for each individual.

## CONFLICT OF INTEREST STATEMENT

The authors have no conflicts of interest to declare.

## Supporting information


**Figure S1:** Model‐estimated (ID‐adjusted) adaptive behaviour v‐scaled score means and 95% confidence intervals for communication domain, daily living domain, socialization domain, and motor domain.

**Table S1:** Vineland Adaptive Behaviour Scales, Third Edition domain and subdomain scores for each monogenic condition.

**Table S2:** Model fit for 2 through 7 cluster models.

## Data Availability

The data that support the findings of this study are available from the corresponding author upon reasonable request.
