# Supplementary material for: Adaptive functioning in children and young adults with monogenic neurodevelopmental disorders
Source: Dev Med Child Neurol. 2025 Jan 23;67(7):953–62. doi: 10.1111/dmcn.16227 (PMC12134409; doi:10.1111/dmcn.16227)
Supplement: Supplementary file 1 — Figure S1: Model‐estimated (ID‐adjusted) adaptive behaviour v‐scaled score means and 95% confidence intervals for communication domain, daily living domain, socialization domain, and motor domain. [file DMCN-67-953-s001.pdf]

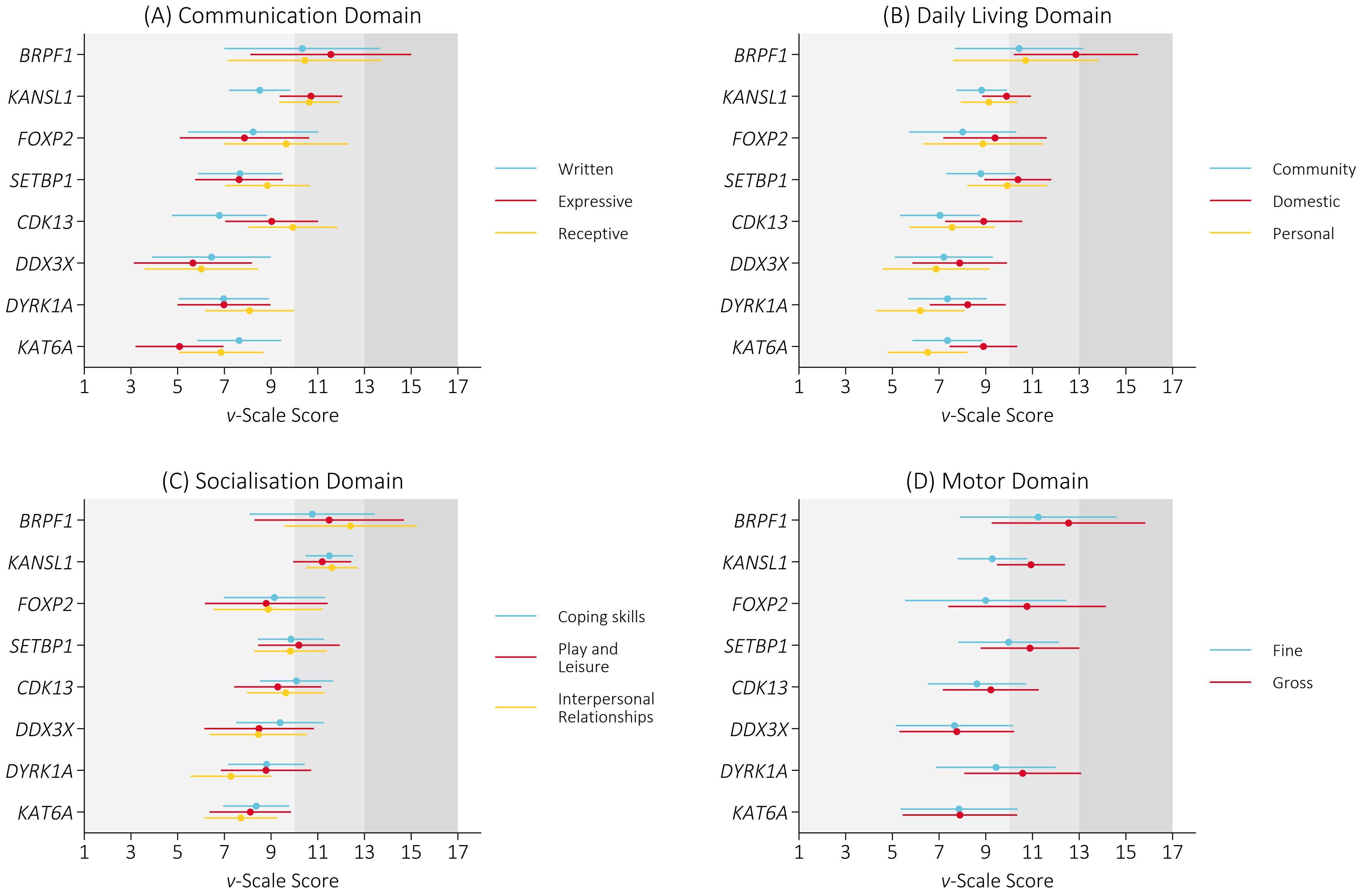

Supplementary Figure S1. Model estimated (ID adjusted) adaptive behaviour v-scaled score means and 95% confidence intervals for (A) Communication Domain; (B) Daily Living Domain; (C) Socialisation Domain; and (D) Motor Domain.
