# Supplementary material for: Adaptive functioning in children and young adults with monogenic neurodevelopmental disorders
Source: Dev Med Child Neurol. 2025 Jan 23;67(7):953–62. doi: 10.1111/dmcn.16227 (PMC12134409; doi:10.1111/dmcn.16227)
Supplement: Supplementary file 2 — Table S1: Vineland Adaptive Behaviour Scales, Third Edition domain and subdomain scores for each monogenic condition. [file DMCN-67-953-s002.docx]

Supplementary Table S1

Vineland Adaptive Behavior Scales Domain and Sub-Domain scores for each Monogenic Condition

|  | ***BRPF1*** | | | ***KANSL1*** | | | ***FOXP2*** | | | ***SETBP1*** | | | ***CDK13*** | | | ***DDX3X*** | | | ***DYRK1A*** | | | ***KAT6A*** | | |
| --- | --- | --- | --- | --- | --- | --- | --- | --- | --- | --- | --- | --- | --- | --- | --- | --- | --- | --- | --- | --- | --- | --- | --- | --- |
|  | n | *M* | (*SD*) | n | *M* | (*SD*) | n | *M* | (*SD*) | n | *M* | (*SD*) | n | *M* | (*SD*) | n | *M* | (*SD*) | n | *M* | (*SD*) | n | *M* | (*SD*) |
| **Adaptive Behaviour Composite (ABC)** | 11 | 87.4 | (14.8) | 66 | 71.7 | (10.4) | 17 | 69.7 | (11.5) | 33 | 68.9 | (10.5) | 32 | 68.7 | (20.3) | 19 | 58.7 | (13.0) | 30 | 57.2 | (17.4) | 30 | 56.8 | (15.1) |
| **Communication Domain** | 11 | 86.0 | (13.4) | 66 | 70.4 | (15.2) | 17 | 70.7 | (15.2) | 33 | 61.8 | (16.9) | 32 | 70.9 | (20.9) | 19 | 46.8 | (18.2) | 30 | 52.8 | (20.3) | 34 | 50.5 | (22.1) |
| Receptive | 11 | 11.9 | (2.9) | 66 | 10.4 | (3.5) | 17 | 10.8 | (3.7) | 33 | 8.8 | (3.5) | 32 | 10.7 | (3.9) | 19 | 5.4 | (4.4) | 31 | 7.5 | (4.5) | 34 | 6.4 | (4.4) |
| Expressive | 11 | 13.3 | (2.8) | 66 | 10.5 | (3.7) | 17 | 9.2 | (4.0) | 33 | 7.6 | (4.2) | 32 | 9.9 | (4.1) | 19 | 4.9 | (4.0) | 31 | 6.3 | (4.3) | 34 | 4.6 | (4.7) |
| Written | 10 | 12.4 | (2.8) | 59 | 8.3 | (3.6) | 14 | 9.7 | (3.2) | 31 | 7.6 | (3.7) | 26 | 7.9 | (5.2) | 16 | 5.6 | (2.2) | 28 | 6.2 | (3.5) | 32 | 7.1 | (4.7) |
| **Daily Living Domain** | 11 | 87.6 | (13.5) | 66 | 67.2 | (12.6) | 17 | 68.7 | (14.8) | 33 | 71.6 | (14.6) | 32 | 65.8 | (19.9) | 19 | 57.5 | (14.6) | 30 | 54.4 | (18.6) | 34 | 58.3 | (16.9) |
| Personal | 11 | 12.0 | (2.7) | 66 | 9.0 | (3.1) | 16 | 9.9 | (3.0) | 33 | 9.9 | (3.5) | 31 | 8.2 | (4.5) | 19 | 6.3 | (4.1) | 28 | 5.7 | (3.5) | 34 | 6.1 | (4.2) |
| Domestic | 10 | 13.6 | (2.2) | 59 | 9.8 | (2.4) | 14 | 9.9 | (3.2) | 31 | 10.4 | (3.3) | 25 | 9.3 | (3.7) | 16 | 7.6 | (1.9) | 25 | 8.0 | (3.2) | 31 | 8.7 | (3.1) |
| Community | 10 | 12.2 | (2.9) | 59 | 8.6 | (2.5) | 14 | 9.3 | (3.0) | 31 | 8.7 | (3.0) | 25 | 7.9 | (4.4) | 16 | 6.4 | (2.7) | 25 | 6.7 | (3.9) | 31 | 6.9 | (3.3) |
| **Socialisation Domain** | 11 | 88.5 | (14.3) | 66 | 78.9 | (14.3) | 17 | 71.1 | (18.2) | 33 | 72.2 | (15.3) | 32 | 73.6 | (19.7) | 19 | 63.4 | (16.8) | 30 | 59.3 | (20.9) | 34 | 60.6 | (19.5) |
| Interpersonal Relationships | 11 | 13.5 | (2.6) | 66 | 11.5 | (2.8) | 16 | 9.7 | (3.1) | 33 | 9.8 | (2.9) | 31 | 10.1 | (3.8) | 19 | 8.0 | (3.2) | 28 | 6.9 | (4.0) | 34 | 7.4 | (3.8) |
| Play and Leisure | 11 | 12.7 | (2.9) | 66 | 11.0 | (3.3) | 16 | 9.8 | (4.7) | 33 | 10.2 | (3.0) | 31 | 9.9 | (4.2) | 19 | 7.9 | (3.9) | 28 | 8.3 | (4.1) | 34 | 7.8 | (4.0) |
| Adapting/Coping | 10 | 11.8 | (2.3) | 64 | 11.4 | (2.7) | 15 | 9.9 | (3.3) | 32 | 9.8 | (2.7) | 28 | 10.6 | (3.5) | 19 | 8.9 | (2.3) | 25 | 8.4 | (3.7) | 33 | 8.1 | (3.1) |
| **Motor Domain** | 9 | 90.6 | (14.6) | 40 | 73.1 | (11.1) | 8 | 77.8 | (29.1) | 20 | 74.9 | (12.8) | 21 | 70.2 | (16.5) | 15 | 56.5 | (18.4) | 16 | 67.9 | (17.8) | 15 | 55.7 | (22.6) |
| Gross Motor | 9 | 14.0 | (3.4) | 40 | 10.8 | (2.5) | 8 | 11.9 | (5.4) | 19 | 11.1 | (3.2) | 21 | 9.8 | (3.6) | 15 | 7.0 | (3.7) | 14 | 10.0 | (2.8) | 15 | 7.1 | (4.7) |
| Fine Motor | 9 | 12.9 | (3.2) | 40 | 9.1 | (2.8) | 8 | 10.2 | (5.3) | 19 | 10.2 | (2.7) | 21 | 9.2 | (3.3) | 15 | 6.8 | (3.9) | 14 | 8.8 | (3.1) | 15 | 7.0 | (5.3) |
