# Supplementary material for: Adaptive functioning in children and young adults with monogenic neurodevelopmental disorders
Source: Dev Med Child Neurol. 2025 Jan 23;67(7):953–62. doi: 10.1111/dmcn.16227 (PMC12134409; doi:10.1111/dmcn.16227)
Supplement: Supplementary file 3 — Table S2: Model fit for 2 through 7 cluster models. [file DMCN-67-953-s003.docx]

Supplementary Table S2

Model fit for 2 through 7 cluster models

| *k* | LL | df | AIC | BIC | BIC Change | LMR | p | Entropy |
| --- | --- | --- | --- | --- | --- | --- | --- | --- |
| 2 | -5142.41 | 28 | 10340.83 | 10438.63 | - | - | - | 0.97 |
| 3 | -4943.50 | 38 | 9963.00 | 10095.74 | 342.90 | 375.08 | <0.001 | 0.95 |
| 4 | -4868.93 | 48 | 9833.86 | 10001.53 | 94.21 | 140.61 | <0.001 | 0.92 |
| **5** | **-4818.75** | **58** | **9753.50** | **9956.10** | **45.43** | **94.62** | **<0.001** | **0.91** |
| 6 | -4790.88 | 68 | 9717.76 | 9955.29 | 0.81 | 52.56 | <0.001 | 0.89 |
| 7 | -4775.97 | 78 | 9707.95 | 9980.41 | -25.12 | 28.11 | 0.081 | 0.89 |

*k*=number of clusters, LL=log-likelihood, df=Degrees of freedom, AIC=Akaike’s Bayesian Information Criteria, BIC=Information Criteria, BIC Change=BIC difference between *k* and *k*-1, LMR=Lo-Mendell-Rubin likelihood ratio test, p=LMR p-value
